# O Efeito Anti-Hipertensivo de *Sauromatum Guttatum* Mediado por Efeitos Vasorrelaxante e Depressivos Miocárdicos

**DOI:** 10.36660/abc.20200055

**Published:** 2021-11-22

**Authors:** Rabia Bibi, Umme Salma, Kashif Bashir, Taous Khan, Abdul Jabbar Shah

**Affiliations:** 1 COMSATS University Islamabad Department of Pharmacy Abbottabad Paquistão COMSATS University Islamabad - Abbottabad Campus - Department of Pharmacy, Abbottabad, Khyber Pakhtunkhwa – Paquistão; 2 Ibadat International University Islamabad Islamabad Paquistão Ibadat International University Islamabad, Islamabad - Paquistão

**Keywords:** Ratos, Anti-Hipertensivos, *Sauromatum guttatum*, Pressão Arterial, Hipertensão, Vasodilatação, Bloqueio do canal de Ca^2+^, Desempenho cardíaco

## Abstract

**Fundamento:**

A *Sauromatum guttatum* (*S. guttatum*) é utilizado no tratamento de doenças do sangue e supostamente tem atividade espasmolítica através da inibição dos canais de Ca^2+^.

**Objetivos:**

O objetivo deste estudo foi investigar o potencial anti-hipertensivo de *S. guttatum* em modelo de rato Sprague-Dawley (SD) com hipertensão induzida por dieta com alto teor de sal (HIDATS).

**Métodos:**

Ratos SD foram divididos em normotensos, hipertensos e grupos tratados com verapamil e *S. guttatum*. Extrato bruto de *S. guttatum* (Sg.B) (100, 150 e 300 mg/kg/dia) e verapamil (5, 10 e 15 mg/kg/dia) foram administrados por via oral junto com NaCl. Anéis aórticos e faixas do átrio direito de ratos normotensos foram utilizados para investigar os mecanismos subjacentes. O nível de significância estatística adotado foi de 5%.

**Resultados:**

A pressão arterial média diminuiu nos grupos hipertensos tratados com Sg.B e verapamil de forma dose-dependente (p <0,001). No estudo de reatividade vascular, a acetilcolina induziu relaxamentos com valor CE_50_ de 0,6 µg/mL (0,3–1,0) em ratos hipertensos tratados com Sg.B (300 mg/kg), sugerindo preservação endotelial. Em aorta isolada de rato normotenso, o Sg.B exibiu vasorrelaxamento com valor de CE_50_ de 0,15 mg/mL (0,10-0,20), após ablação por desnudamento endotelial ou pré-tratamento com L-NAME e atropina. O tratamento com Sg.B causou relaxamento contra contrações induzidas por K^+^ alto, como o verapamil. O Sg.B mostrou efeitos inotrópicos (82%) e cronotrópicos (56%) negativos em preparações isoladas atriais de ratos reduzidas com atropina. A avaliação fitoquímica indicou a presença de alcaloides, flavonoides e taninos.

**Conclusão:**

O *S. guttatum* possui efeito vasodilatador através da preservação da função endotelial, liberação de NO mediada pelo receptor muscarínico e inibição do movimento de Ca^2+^, enquanto o efeito depressor do miocárdio atrial pode estar ligado ao receptor muscarínico. Esses achados fornecem a base farmacológica para o uso do extrato de *S. guttatum* como um medicamento anti-hipertensivo.

## Introdução

A hipertensão é um fator de risco importante para doenças cardiovasculares e mortalidade devido aos danos em órgãosalvo.^1^ Existem muitos fatores ambientais que contribuem para a etiologia da hipertensão, incluindo ingestão elevada de sal. A alta ingestão de sal continua sendo o fator mais importante na etiologia da hipertensão em humanos.^2^ Em ratos, a alta ingestão de sal também promove a hipertensão, fornecendo um modelo conveniente para estudar a hipertensão humana.^3^ O consumo contínuo de uma dieta rica em sal leva à disfunção endotelial, o que pode representar um fator de risco particularmente significativo no desenvolvimento da hipertensão,^4^ afetando negativamente a qualidade de vida.^5^ As medidas para o manejo da hipertensão incluem ajuste no estilo de vida, modificação da dieta, exercícios físicos, bem como terapias convencionais e alternativas, incluindo remédios fitoterápicos.^6-8^ A *Sauromatum guttatum* (*S. guttatum*) pertence à família Araceae, e é comumente conhecida como “lírio vodu” e “monarca do Oriente”. A *Sauromatum guttatum* é conhecida como “*Sanp ki Booti*” no Paquistão e na Índia, onde é onipresente. A *S. guttatum* é tradicionalmente utilizada para tratar inflamação, dificuldades respiratórias,^9^ problemas gástricos,^10^ tuberculose, doenças do sangue, picadas de cobra e infecções de pele.^11^ A *S. guttatum* contém lectinas, dimetilsulfetos, cariofileno, indol, escatol, amônia, trimetilamina e aminas primárias.^12-14^ Os cormos ou bulbos contêm carbono, magnésio, enxofre, oxigênio, fósforo, potássio e cloro. Estudos *in vitro* revelaram atividades mitogênica,^15^ antiproliferativa,^16^ herbicida,^17^ inibidora da lipoxigenase,^18^ antioxidante, antibacteriana,^19^ espasmolítica^18,20^ e inseticida da *S. guttatum*.^17,21^ É tradicionalmente utilizada para o tratamento de doenças do sangue. Foi relatado anteriormente que seu efeito espasmolítico é mediado pelo bloqueio de entrada de Ca^2+^ na musculatura lisa do intestino.^20^ Os bloqueadores de entrada de Ca^2+^ também têm papel terapêutico importante no tratamento da hipertensão. Todas essas observações fornecem uma base sólida para nossa hipótese de que o extrato de *S. guttatum* pode ter propriedades anti-hipertensivas. O objetivo deste estudo foi investigar o potencial anti-hipertensivo da *S. guttatum* e revelar os mecanismos subjacentes utilizando métodos *in vivo* e *in vitro*.

## Materiais e métodos

### Preparação do extrato bruto e análise fitoquímica

Cormos de *S. guttatum* foram adquiridos em Nathia Gali, Paquistão (junho-julho de 2018), identificados e validados pelo Dr. Abdul Nazir, Professor Assistente do Departamento de Biotecnologia, COMSATS University Islamabad, Abbottabad Campus, Paquistão. CHUA-112 é o código de voucher para o espécime no herbário do Departamento de Farmácia, COMSATS University Islamabad, Abbottabad Campus, Paquistão. Cormos frescos foram picados e submetidos à secagem na sombra em temperatura ambiente. Em seguida, o material seco foi pulverizado, mergulhado em metanol aquoso (70%) com agitação ocasional durante quinze, sete e três dias. O macerado foi filtrado em um tecido de musselina e, em seguida, com um papel filtro qualitativo (Whatman, Grau 1).^22^ Esse processo foi repetido três vezes. Em seguida, um evaporador rotativo (-760 mmHg a 37°C) foi utilizado para concentrar o extrato líquido. O extrato bruto foi analisado fitoquimicamente para todos os constituintes importantes, como flavonoides, alcaloides, saponinas, fenóis e taninos.^23^

### Animais

Todos os experimentos foram realizados em conformidade com as orientações da *Commission on Life Sciences, Institute of Laboratory Animal Resources, National Research Council* ^24^ e aprovadas pelo Comitê de Ética. Os ratos Sprague-Dawley (SD) foram mantidos no Biotério com comida e água disponíveis *ad libitum*.

### Investigações farmacológicas

#### Medicamentos e padrões

Os medicamentos e padrões foram adquiridos das seguintes fontes: cloreto de acetilcolina, cloridrato de fenilefrina, sulfato de atropina e o pentotal sódico dos Laboratórios Abbott, Paquistão; o cloridrato de isoprenalina, cloreto de potássio, cloridrato de éster metílico de Nω-Nitro-L-arginina (L-NAME) e cloridrato de verapamil da empresa Sigma Chemicals, EUA.

#### Estudos *in vivo*

##### Modelo e grupos de ratos com hipertensão induzida por dieta com alto teor de sal (HIDATS)

Ratos SD (200-250 g) (n = 60) foram divididos aleatoriamente em oito grupos (n = 5-7 em cada grupo). A amostragem foi feita por conveniência. O Grupo 1 (grupo controle normal) recebeu dieta normal. O Grupo 2 (grupo de hipertensos) recebeu NaCl (8% na dieta + 1% na água de beber) por 8 semanas. Os grupos 3-5 (grupo tratado com *S. guttatum*) receberam Na Cl (8% na dieta + 1% na água potável) e diferentes doses orais de extrato bruto de *S. guttatum* (100 mg/kg/dia, 150 mg/kg/dia e 300 mg/kg/dia) uma vez por dia durante 8 semanas. Os grupos 6 a 8 (grupo com tratamento padrão) receberam por via oral doses diárias de verapamil (5 mg/kg/dia, 10 mg/kg e 15 mg/kg/dia) junto com dieta de NaCl contendo 8% NaCl + 1% NaCl na água de beber por 8 semanas.^25-27^

##### Registro invasivo de pressão arterial em ratos com HIDATS

A intubação traqueal de ratos SD anestesiados (pentotal, 40–100 mg/kg, IP) foi realizada com tubo de polietileno (PE-20). Para monitorar a pressão arterial, a artéria carótida direita foi canulada com tubo de polietileno (PE-50) e afixada no *PowerLab Data Acquisition System* (ADInstrument, Austrália), através de um transdutor de pressão (MLT 0699). Uma lâmpada suspensa foi utilizada para manter a temperatura corporal do animal. A pressão arterial média foi monitorada por 30 minutos em cada grupo.^25,28^

##### Perfil de peso corporal

O peso corporal de todos os grupos foi determinado no início do experimento e posteriormente monitorado semanalmente. Após 8 semanas de tratamento, a mudança no peso corporal foi calculada.

#### Estudos *in vitro*

##### Estudos de reatividade vascular

Para investigar o efeito da preservação do endotélio induzido pelo extrato bruto em ratos com HIDATS, isolamos a aorta dos grupos normotenso, hipertenso e tratados. Os anéis aórticos foram suspensos em banhos teciduais (10 mL), contendo carbogênio (5% CO_2_ e 95% O_2_) solução de Krebs normal aerada, composta por NaCl, 118,2 mM; KCl, 4,7 mM; MgSO_4_, 1,2 mM; KH_2_PO_4_, 1,3 mM; NaHCO_3_, 25,0 mM; Glicose, 11,7 mM; CaCl_2_, 2,5 mM, mantido a 37ºC. A força foi monitorada pelo *PowerLab Data Acquisition System* (ADInstrument, Austrália) e um amplificador em ponte (N12128) através de um transdutor de deslocamento de força (MLT 0201). Os anéis aórticos foram estabilizados em tensão isométrica a 2 g por 60-90 minutos, com troca da solução de Krebs a cada 15 minutos. Para determinar a integridade do endotélio, diferentes concentrações de acetilcolina foram utilizadas em anéis aórticos pré-constritos com fenilefrina (1 µM).^25,28^

##### Preparações isoladas de aorta de rato SD

Os anéis aórticos foram suspensos em banhos teciduais contendo 10 mL de carbogênio (5% CO_2_ e 95% O_2_) e solução de Krebs normal aerada mantida a 37°C, fixada no *PowerLab Data Acquisition System* (ADInstrument, Austrália) e um amplificador em ponte (N12128) através de um transdutor de deslocamento de força (MLT 0201). Os anéis foram equilibrados por 60-90 minutos a uma tensão isométrica de 2 g, enquanto a solução foi trocada a cada 15 minutos. Diferentes concentrações (0,1–10 mg/mL) de *S. guttatum* foram adicionadas aos anéis pré-constritos com fenilefrina (FE). Para determinar o mecanismo subjacente, os anéis aórticos foram pré-tratados com atropina 1 µM ou L-NAME 10 µM por 30 minutos. Em alguns experimentos, anéis desnudados de endotélio de ratos normotensos foram utilizados.^25,28,29^

##### Preparações isoladas do átrio direito

Os átrios direitos de ratos SD normotensos foram dissecados. As preparações atriais foram suspensas em banhos teciduais contendo 10 mL de solução aerada de Krebs, mantida a 32°C, ligada ao *PowerLab (ML 846) Data Acquisition System* (ADInstrument, Austrália) e amplificador em ponte (N12128), através de um transdutor de força (MLT 0201). Os tecidos foram estabilizados em uma tensão de repouso de 1 g por 30 minutos. O envolvimento do receptor muscarínico foi estudado em preparações atriais pré-tratadas com atropina (1 *µ*M).^25,28^

##### Análise estatística

Os dados tinham distribuição normal, como determinado pelo teste de normalidade de Shapiro-Wilk. Os dados foram expressos como média ± desvio padrão (DP) e as medianas das concentrações efetivas (valores de CE_50_) com intervalo de confiança (IC) de 95%. A porcentagem de mudança nos perfis de pressão arterial média (PAM) ou peso corporal foram calculados por análise de variância (ANOVA) de uma via (seguida por teste *post-hoc* de Tukey HSD). A porcentagem de vasorrelaxamento em ratos normotensos e hipertensos foi calculada por ANOVA de duas vias (seguida pelo teste *post-hoc* de Bonferroni) utilizando o software SPSS 21 (EUA). O nível de significância estatística aceito foi de 5%.

## Resultados

### Constituintes fitoquímicos

A análise fitoquímica preliminar realizada no extrato de cormos de *S. guttatum* identificou alcaloides, flavonoides, fenóis, fitosteróis, saponinas e taninos.

#### Investigação farmacológica

#### Estudos *in vivo*

##### Monitoramento invasivo de pressão arterial

**Figura 1:**
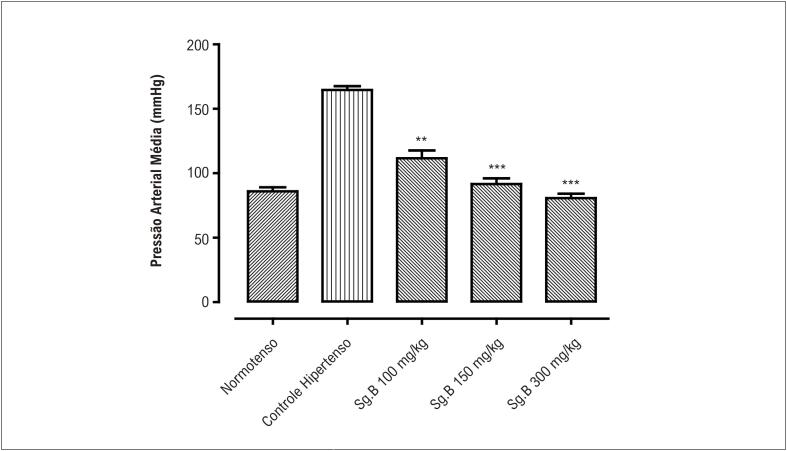
Pressão arterial média em ratos normotensos, hipertensos e ratos tratados com extrato bruto de Sauromatum guttatum (Sg.Cr) para hipertensão induzida por dieta com alto tear de sal, em doses de 100 mg/kg, 150 mg/kg e 300 mg/kg (n = 5- 7; média ± EPM). Comparado com os valores do grupo controle hipertensivo, **p <0,01 e ***p <0,001

Os valores da pressão arterial média (PAM) medidos nos diferentes grupos experimentais são mostrados nas Figuras 1 e 2. A PAM do grupo de ratos com HIDATS apresentou elevação de 67,7% da pressão arterial em comparação ao grupo controle normotenso. Esta elevação na PAM foi revertida pelo tratamento com Sg.B de maneira dose-dependente (p <0,01) até a concentração de 300 mg/kg, onde atingiu seu efeito máximo de redução da PAM (p<0,001). A PAM em ratos tratados com verapamil (5 mg/kg e 10 mg/kg) também diminuiu de maneira dose-dependente (p<0,01 e p<0,001 respectivamente) atingindo o efeito máximo na concentração de 15 mg/kg (p<0,001).

**Figura 2:**
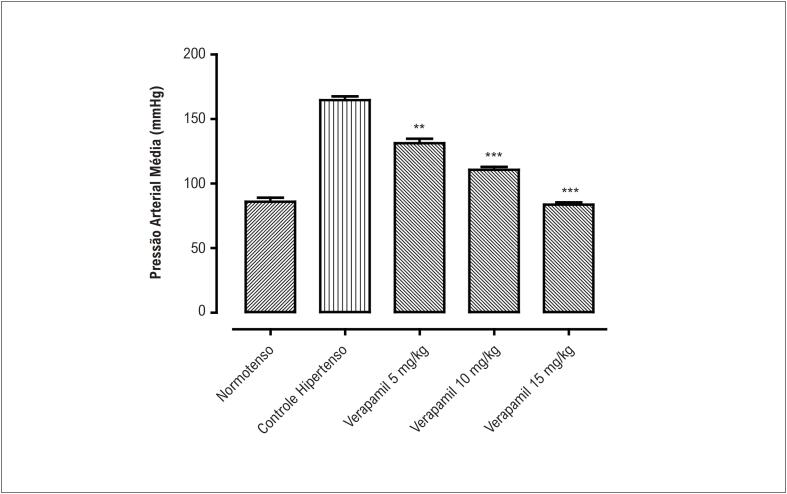
Pressão arterial média em ratos normotensos, hipertensos e com hipertensão induzida por dieta com alto teor de sal tratados com verapamil nas doses de 5 mg/kg, 10 mg/kg e 15 mg/kg) (n = 5-7; média ± EPM). Comparado com os valores do grupo controle hipertenso, **p<0,01 e ***p<0,001

##### Perfil de peso corporal

A alta ingestão de sal por 8 semanas causou uma diminuição significativa (p < 0,001) no peso corporal no grupo controle hipertenso (Tabela 1). O tratamento de ratos hipertensos com extrato bruto de *Sauromatum guttatum* (Sg.B) evitou alteração significativa no peso corporal em todas as doses, enquanto os animais tratados com verapamil na dose de 5 mg/kg apresentaram redução significativa no peso corporal (p < 0,05). Os animais nos grupos tratados com verapamil (ambos, 10 mg/kg e 15 mg/kg) não apresentaram alteração significativa no peso corporal (Tabela 1).

**Tabela 1:** Efeito no peso corporal dos grupos controle normal, controle hipertenso e ratos tratados com diferentes doses do extrato bruto de Sauromatum guttatum (Sg.B) e verapamil. Os valores sao expressos como média ± DP (n = 5-7).

Grupos	Peso (g)	Peso (g) após 8 semanas
Controle normal	244,66 ± 6,36	267,61 ± 3,08
Grupo hipertenso	249,28 ± 3,25	182,10 ±5,09***
Tratado com Sg.B 100 mg/kg	241,66 ±3,81	245,01 ± 4,66
Tratado com Sg.B 150 mg/kg	245,93 ± 6,43	250,90 ± 3,53
Tratado com Sg.B 300 mg/kg	239,43 ± 1,48	248,63 ± 4,52
Tratado com Verapamil 5 mg/kg	240,50 ± 1,41	214,23 ± 3,53*
Tratado com Verapamil 10 mg/kg	242,25 ± 5,65	247,68 ± 2,96
Tratado com Verapamil 15 mg/kg	245,83 ± 6,36	254,48 ± 3,32

Sg.B: Extrato bruto de Sauromatum guttatum. Os valores são expressos como média ± DP (n = 5-7).

* p<0,05,

** p<0,01 e

*** p<0,001

vs. valores de pré-tratamento (análisc ANOVA de uma via seguida de teste post-hoc de Tukcy HSD)

#### Estudos i*n vitro*

##### Estudos de reatividade vascular *in vitro*

**Figura 3:**
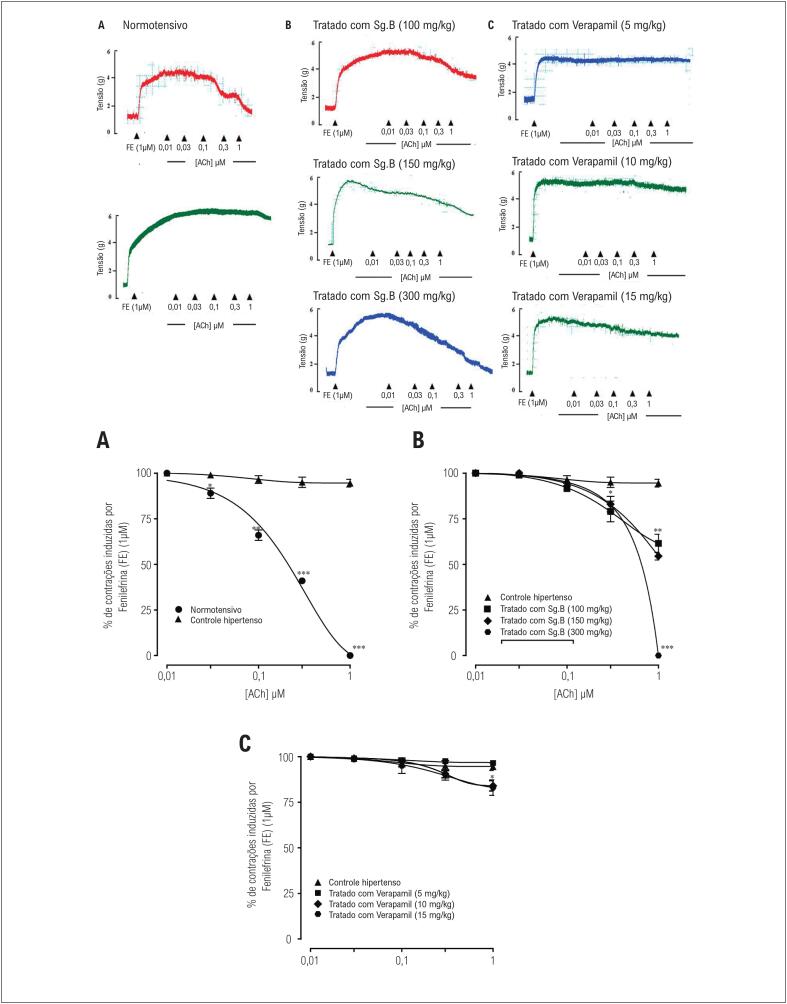
Traçados e gráficos típicos mostram o efeito da acetilcolina (ACh) contra as contrações induzidas por fenilefrina nos anéis aórticos isolados de ratos normotensos, grupo controle hipertenso (A) e de ratos tratados com extrato bruto de Sauromatum guttatum (Sg.Cr) com hipertensão induzida por dieta com alto teor de sal, em doses de 100 mg/kg, 150 mg/kg e 300 mg/kg (B) e em ratos tratados com verapamil com hipertensão induzida por dieta com alto teor de sal em doses de 5 mg/kg, 10 mg/kg e 15 mg/kg (C) (n = 5-7; média ± DP). Em comparação com os valores do grupo controle hipertenso, *p <0,05, **p <0,01 e ***p <0,001

Em aortas isoladas do grupo normotenso, a acetilcolina causou relaxamento completo com valor de CE_50_ de 0,2 µM (0,1–0,3) (Figura 3). Por outro lado, as aortas de ratos do grupo controle hipertenso exibiram apenas 5,5% de relaxamento dependente de acetilcolina, como mostrado na Figura 3. O tratamento com extrato bruto de *S. guttatum* de 100 mg/kg e 150 mg/kg restaurou parcialmente o relaxamento induzido por acetilcolina para 38,5 % e 45,5%, respectivamente. Entretanto, os anéis de ratos SD tratados com 300 mg/kg de extrato bruto de *S. guttatum* mostraram 100% de relaxamento dependente de acetilcolina, com valor de CE_50_ de 0,6 µM (0,3-1,0) (Figura 3). O tratamento com verapamil 5 mg/kg causou apenas um relaxamento insignificante, enquanto o tratamento com 10 mg/kg induziu relaxamento de até 16%. Curiosamente, o aumento da concentração de verapamil para 15 mg/kg não aumentou ainda mais o relaxamento induzido pela acetilcolina (Figura 3).

##### Estudos *in vitro* da aorta de rato

**Figura 4:**
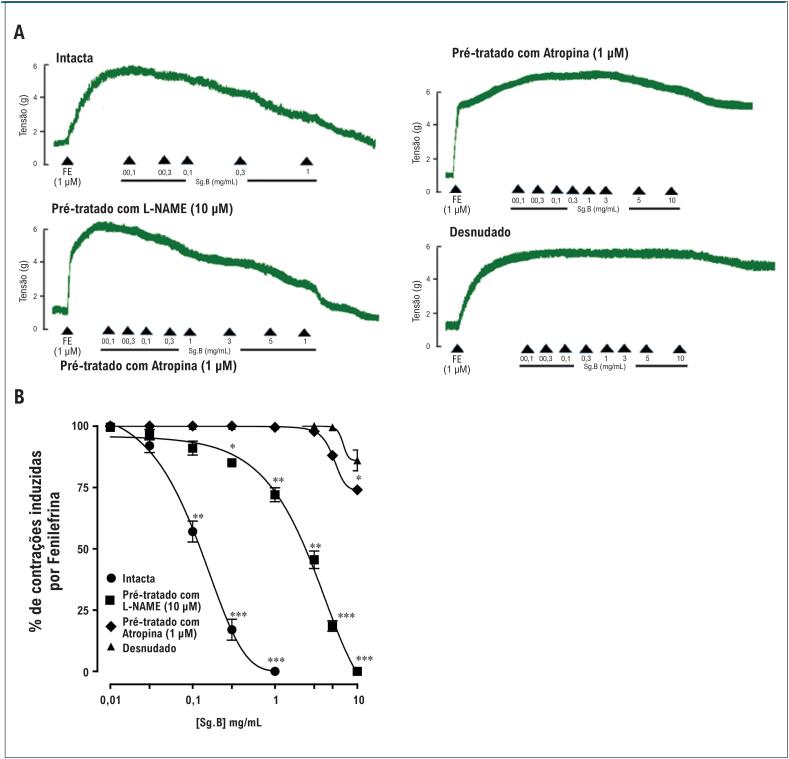
O traçado (A) e o gráfico (B) mostram o efeito do extrato bruto de Sauromatum guttatum em aorta intacta, pré-tratada com L-NAME (10 *µ*M) e atropina (1 *µ*M) e em aorta de rato normotenso com endotélio desnudado versus contrações induzidas por fenilefrina (n = 5-7; média ± DP). *p<0,05, **p<0,01 e ***p<0,001 vs. Controle (valores pré-tratados). Análise ANOVA de duas vias seguida pelo teste post-hoc de Bonferroni.

**Figura 5:**
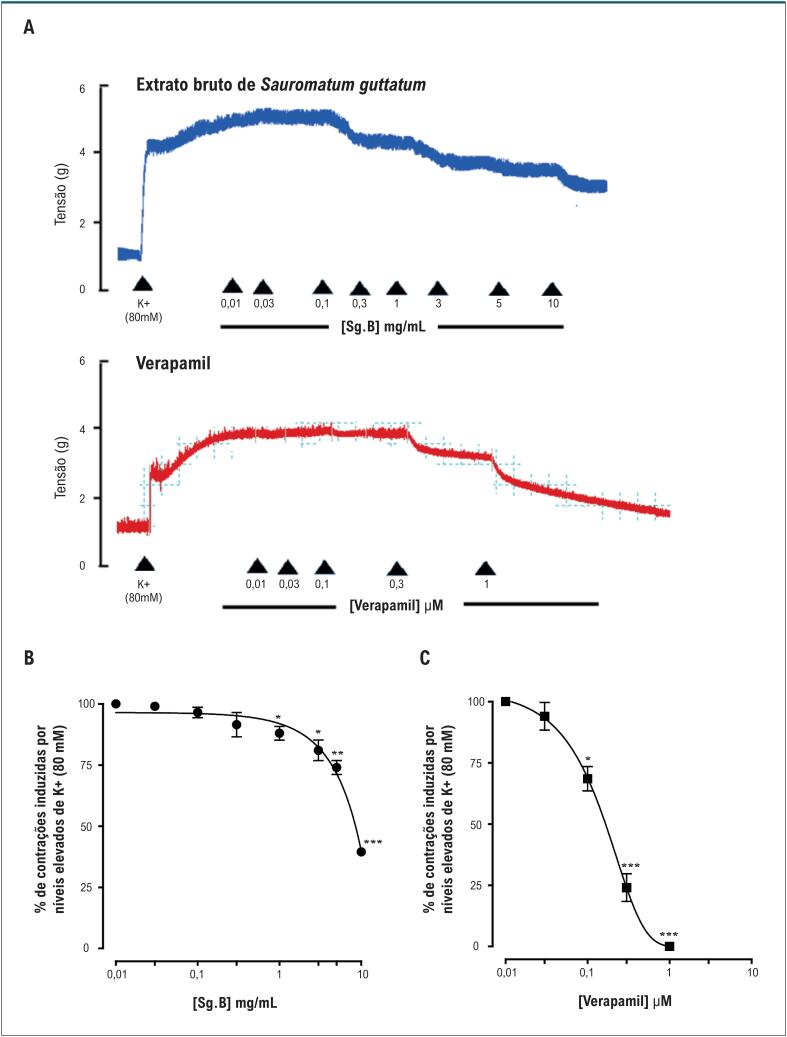
O traçado (A) e os gráficos (B, C) mostram o efeito do extrato bruto de Sauromatum guttatum nas contrações induzidas por potássio elevado (K^+^) (80 mM) na preparação da aorta intacta de rato (n = 5-7; média ± DP). *p<0,05, **p<0,01 e ***p<0,001 vs. Controle (valores pré-tratados). Análise ANOVA de duas vias seguida pelo teste post-hoc de Bonferroni.

Estudos farmacológicos foram realizados na aorta de ratos normotensos para investigar o efeito anti-hipertensivo do extrato bruto *S. guttatum*. Relaxamentos induzidos pela adição cumulativa de extrato bruto em anéis aórticos pré-constritos com FE apresentaram um valor de CE_50_ de 0,15 mg/mL (0,10-0,20) (Figura 4). Os anéis pré-tratados com L-NAME (10 *µ*M) mostraram relaxamento com valor de CE_50_ de 5,1 mg/mL (3,0-7,1) (Figura 4). O extrato bruto de *S. guttatum* não foi capaz de induzir relaxamento em anéis pré-tratados com atropina (1 *µ*M) e anéis desnudados de endotélio. O pré-tratamento com atropina (1 *µ*M) e a remoção do endotélio diminuíram o relaxamento induzido pelo extrato bruto em 26% e 14%, respectivamente (Figura 4). O extrato bruto de *S. guttatum* também produziu vasorrelaxamento em anéis aórticos pré-constritos com alta concentração de K^+^ com valor de CE_50_ de 9,03 mg/mL (8,06-10,00). Em comparação, o verapamil relaxou as aortas pré-constritas com níveis altos de K^+^ com valor de CE_50_ de 2,02 *µ*M (1,02-3,02) (Figura 5).

##### Estudo *in vitro* do átrio direito em ratos

**Figura 6:**
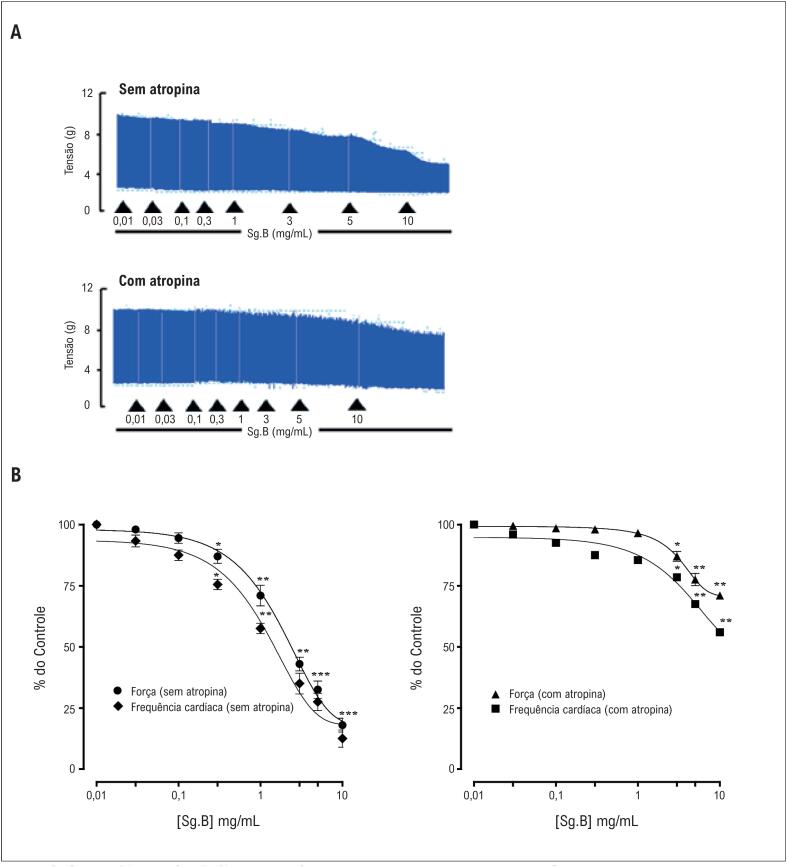
O traçado (A) e os gráficos (B, C) mostram os efeitos inotrópico e cronotrópico do extrato bruto de Sauromatum guttatum sem e com atropina (1 *µ*M) no átrio direito de ratos normotensos pré-tratados (n = 5-7; média ± DP) *p <0,05, ** p <0,01 e ***p <0,001 vs. controle (valores pré-tratados). Análise ANOVA de duas vias seguida pelo teste post-hoc de Bonferroni

Tiras do átrio direito de ratos normotensos foram utilizadas para investigar os efeitos cronotrópicos e inotrópicos de *S. guttatum*. O extrato bruto mostrou uma diminuição dose-dependente na força de contração e na frequência cardíaca com valor de CE_50_ de 2,99 mg/mL (1,08-4,90) e 1,83 (1,02-2,64), respectivamente (Figura 6). Em tecidos pré-tratados com atropina, a diminuição da força de contração e da frequência cardíaca foi de 29% e 44%, respectivamente (Figura 6).

## Discussão

A *S. guttatum* tem sido tradicionalmente utilizada no tratamento de doenças do sangue. Ela contém uma grande quantidade de magnésio e potássio.^11,15^ Além disso, seu efeito antioxidante, espasmolítico e como bloqueador de entrada de Ca^2+^ já foi relatado.^19,20^

Plantas com propriedades antioxidantes, a dieta DASH (*Dietary Approaches to Stop Hypertension*.) rica em potássio e magnésio e bloqueadores de entrada de Ca^2+^ são recomendados para o manejo da hipertensão.^8,30,31^ O presente estudo, utilizando o modelo de rato SD hipertenso, foi realizado para explorar o extrato bruto de *S. guttatum* como um potencial medicamento anti-hipertensivo. Diferentes doses de extrato bruto de *S. guttatum* foram administradas por via oral a ratos SD com hipertensão induzida por dieta com alto teor de sal. Esse tratamento resultou em diminuição significativa na pressão arterial média, com efeito máximo observado com uma dose de 300 mg/kg. Este efeito do extrato bruto foi comparável ao do verapamil, que é um fármaco anti-hipertensivo padrão e bloqueador dos canais de cálcio.^31^ Esse achado revelou que o extrato de *S. guttatum* é eficaz contra o desenvolvimento de hipertensão experimental induzida por dieta rica em sal. No entanto, são necessários mais estudos para identificar o possível mecanismo de ação subjacente.

Uma vez que a pressão arterial é o produto de elevada resistência vascular periférica e alto débito cardíaco,^32^ outros experimentos foram realizados utilizando preparações vasculares e cardíacas isoladas. Primeiro, foi feita uma tentativa de estabelecer como a alta ingestão de sal induz a disfunção endotelial. A integridade do endotélio foi confirmada pela aplicação de concentrações submáximas de acetilcolina em anéis aórticos pré-constritos com fenilefrina em ratos com HIDATS. A acetilcolina não foi capaz de induzir relaxamento nos anéis aórticos do grupo de ratos com HIDATS, indicando que o endotélio foi danificado. Esse achado é corroborado por estudos anteriores.^33-35^ Em anéis aórticos de ratos normotensos, por outro lado, as mesmas concentrações de acetilcolina induzem relaxamento, indicando a presença de endotélio funcional. Nos grupos tratados com extrato, a resposta à acetilcolina foi restaurada. Esses resultados indicam que o tratamento com extrato bruto pode reverter o dano endotelial e também evitar a elevação da pressão arterial média observada *in vivo*. Em comparação, o verapamil falhou em induzir vasorrelaxamento em anéis aórticos de ratos com HIDATS do grupo controle ou ratos tratados, indicando que seu mecanismo de ação é diferente do extrato bruto. O extrato de *S. guttatum* exerce sua função anti-hipertensiva na hipertensão experimental através da preservação parcial da função endotelial.

Outros estudos *in vitro* foram realizados na aorta para investigar o(s) mecanismo(s) de ação(ões) subjacente(s). Em anéis aórticos pré-constritos com acetilcolina de ratos normotensos, a adição cumulativa de concentrações de extrato bruto induziu vasorrelaxamento. A remoção do endotélio reverteu completamente esse efeito, sugerindo que fatores derivados do endotélio vascular podem desempenhar um papel. No entanto, a alta concentração de acetilcolina ainda induziu relaxamento, sugerindo envolvimento de diferentes mecanismos. Para estudar o envolvimento do óxido nítrico, os anéis aórticos foram pré-tratados com L-NAME, um inibidor de óxido nítrico sintase.^36^ Curiosamente, o efeito vasorrelaxante do extrato de *S. guttatum* foi reduzido em cerca de 75% na concentração de 1 mg/mL, embora em concentrações mais altas ele tenha desviado a curva de resposta para a direita. Esses achados sugerem que o extrato de *S. guttatum* induz vasorrelaxamento através tanto da via dependente do endotélio (em concentração mais baixa) como da via independente do endotélio (em concentração mais alta). O componente dependente do endotélio pode ser atribuído ao óxido nítrico. Em células endoteliais vasculares, a liberação de óxido nítrico é acoplada a receptores muscarínicos.^37^ Para verificar se o efeito do extrato bruto de *S. guttatum* está ligado a receptores muscarínicos e ao óxido nítrico, os anéis aórticos foram contraídos com atropina, um antagonista do receptor muscarínico.^37^ Este pré-tratamento aboliu o vasorrelaxamento associado ao extrato bruto de *S. guttatum*, indicando assim uma ação através da via do óxido nítrico (NO) ligada ao receptor muscarínico. A Atropina ou o L-NAME não foram capazes de inibir o relaxamento em concentrações mais elevadas do extrato bruto, sugerindo ainda que o extrato também pode atuar nos músculos lisos vasculares. Para testar essa hipótese, anéis aórticos foram contraídos com alta concentração de K^+^. Curiosamente, a adição cumulativa do extrato bruto induziu um efeito vasorrelaxante 10 vezes menos potente do que contra a FE. O K^+^ elevado foi utilizado para induzir contrações, pois ativa os canais de cálcio dependentes de voltagem (Cav) e a liberação de Ca^2+^ através da despolarização, resultando em vasoconstrição.^38,39^ Esses achados indicam que o extrato bruto de *S. guttatum* também inibe a entrada de Ca^2+^ pelos canais dependentes da voltagem. Também sugere que o NO vascular desempenha um papel dominante no efeito vasorrelaxante e anti-hipertensivo de *S. guttatum*, além do efeito nos músculos lisos vasculares.

Para investigar o efeito do extrato de *S. guttatum* nos parâmetros cardíacos, foram utilizadas tiras atriais isoladas de ratos. O extrato de *S. guttatum* mostrou efeitos negativos inotrópicos (82%) e cronotrópicos (56%) quando adicionado cumulativamente às tiras do átrio direito que se contraem espontaneamente. Para testar o possível papel dos receptores muscarínicos cardíacos, as tiras atriais foram pré-tratadas com atropina. Este pré-tratamento inibiu parcialmente o efeito do extrato bruto de *S. guttatum*, indicando assim a possibilidade de que o efeito negativo inotrópico ou cronotrópico observado seja devido à ativação de receptores muscarínicos cardíacos. Entretanto, nossos achados revelaram que o extrato é mais seletivo para os receptores muscarínicos vasculares do que para os receptores cardíacos.

O extrato de *S. guttatum* também foi testado quanto à presença de constituintes fitoquímicos. Foi verificado que ele contém flavonoides, fenóis e taninos. Estudos anteriores revelaram o efeito terapêutico de flavonoides, fenóis e taninos na hipertensão.^40-42^ Portanto, esses constituintes podem ser os agentes ativos responsáveis pela redução da pressão arterial e efeitos vasculares na hipertensão induzida por alto teor de sal. Os estudos fitoquímicos futuros serão concentrados no isolamento dos componentes ativos e na exploração dos mecanismos subjacentes, como o bloqueio do cálcio e a via do óxido nítrico a nível molecular.

## Conclusão

Esses achados indicam que *S. guttatum* possui atividade anti-hipertensiva, resultando em efeitos vasodilatadores e depressores do miocárdio atrial ligados a receptores muscarínicos. A preservação da função endotelial, a liberação de NO dependente do receptor muscarínico e a inibição do movimento de Ca^+2^ são os mecanismos subjacentes de vasodilatação. O extrato de *S. guttatum* também exerce efeitos negativos inotrópico e cronotrópico, possivelmente devido à ativação de receptores muscarínicos cardíacos. Nossos resultados, observados no modelo de rato SD, fornecem uma explicação farmacológica para o potencial anti-hipertensivo de *S. guttatum*.
