# Supplementary material for: O Efeito Anti-Hipertensivo de Sauromatum Guttatum Mediado por Efeitos Vasorrelaxante e Depressivos Miocárdicos
Source: Arq Bras Cardiol. 2021 Nov 22;117(6):1093–103. [Article in Portuguese] doi: 10.36660/abc.20200055 (PMC8757165; doi:10.36660/abc.20200055)
Supplement: Translation

# Translation: Antihypertensive Activity of Sauromatum guttatum Mediated by Vasorelaxation and Myocardial Depressant Effects

**Authors:** Rabia Bibi, Umme Salma, Kashif Bashir, Taous Khan, Abdul Jabbar Shah

**Affiliations:** 1 Abbottabad Pakistan COMSATS University Islamabad - Abbottabad Campus - Department of Pharmacy, Abbottabad, Khyber Pakhtunkhwa – Pakistan; 2 Islamabad Pakistan Ibadat International University Islamabad, Islamabad - Pakistan

**Keywords:** Rats, Antihypertensive, Sauromatum guttatum, Blood Pressure, Hypertension, Vasodilatation, Calcium Channel Blockers, Cardiac Performance

## Abstract

**Background:**

Sauromatum guttatum (S. guttatum) is used in the treatment of blood disorders and reportedly has a spasmolytic activity through Ca^2+^ channel inhibition.

**Objectives:**

The aim of this study was to investigate the antihypertensive potential of S. guttatum in high salt-induced hypertensive Sprague-Dawley (SD) rat model (HSHRs).

**Methods:**

SD rats were divided into normotensive, hypertensive, S. guttatum and verapamil treated groups. S. guttatum crude extract (Sg.Cr) (100, 150 and 300 mg/kg/day) and verapamil (5, 10 and 15 mg/kg/day) were administered orally along with NaCl. Aortic rings and right atrial strips from normotensive rats were used to investigate the underlying mechanisms. The level of statistical significance was set at 5%.

**Results:**

Mean arterial pressure decreased in the Sg.Cr and verapamil-treated hypertensive groups in a dose-dependent manner (p < 0.001). In the vascular reactivity study, acetylcholine induced relaxations with an EC_50_ value of 0.6 *µ*g/mL (0.3–1.0) in Sg.Cr-treated hypertensive rats (300 mg/kg), suggesting endothelial preservation. In isolated normotensive rat aorta, Sg.Cr-treated rats showed vasorelaxation with an EC_50_ value of 0.15 mg/mL (0.10-0.20), ablated by endothelial denudation or pretreatment with _L_-NAME and atropine. Sg.Cr treatment caused relaxation against high K^+^-induced contractions, like verapamil. Sg.Cr showed negative inotropic (82%) and chronotropic effects (56%) in isolated rat atrial preparations reduced with atropine. The phytochemical investigation indicated presence of alkaloids, flavonoids and tannins.

**Conclusion:**

S. guttatum has a vasodilatory effect through endothelial function preservation, muscarinic receptor-mediated NO release and Ca^2+^ movement inhibition, while atrial myocardial depressant effect can be linked to the muscarinic receptor. These findings provide pharmacological base for using S. guttatum extract as an antihypertensive medication.

## Introduction

Hypertension is an important risk factor for cardiovascular disease and mortality due to target-organ damage.^1^ There are many environmental factors that contribute to the etiology of hypertension, including high salt intake. High salt intake has remained the most important factor in the etiology of hypertension in humans.^2^ In rats, high salt intake also promotes hypertension, providing a convenient model to study human hypertension.^3^ Sustained consumption of a high-salt diet leads to endothelial dysfunction, which may pose a particularly significant risk factor in the development of hypertension,^4^ negatively affecting the quality of life.^5^ Hypertension management measures include lifestyle adjustments, diet modification, exercise, as well as conventional and alternative therapies, including herbal remedies.^6-8^ *Sauromatum guttatum* (*S. guttatum*) belongs to the Araceae family, and is commonly known as “Voodoo Lilly”. *Sauromatum guttatum* is known as “Sanp ki Booti” in Pakistan and India, where it is ubiquitous. *S. guttatum* is traditionally used for treating inflammation, breathing difficulties,^9^ gastric troubles,^10^ tuberculosis, blood disorders, snakebites and skin infections.^11^ *S. guttatum* contains lectins, dimethyl sulphides, caryophyllene, indole, skatole, ammonia, trimethylamine and primary amines.^12-14^ The corms contain carbon, magnesium, sulfur, oxygen, phosphorus, potassium and chlorine. *In vitro* studies revealed *S. guttatum's* mitogenic,^15^ antiproliferative,^16^ herbicidal,^17^ lipoxygenase inhibitor,^18^ antioxidant, antibacterial,^19^ spasmolytic^18,20^ and insecticidal activities.^17,21^ It is traditionally used to manage blood disorders. It has been previously reported that its spasmolytic effect is mediated through Ca^2+^ entry blockade in the smooth muscles of the intestine.^20^ Ca^2+^ entry blockers also have an important therapeutic role in the management of hypertension. All these observations provide a solid foundation for our hypothesis that *S. guttatum* extract might have antihypertensive properties. The objective of this study was to investigate the antihypertensive potential of *S. guttatum* and to reveal the underlying mechanisms by using *in vivo* and *in vitro* methods.

## Materials and methods

### Preparation of crude extract and phytochemical screening

*S. guttatum* corms were procured in Nathia Gali, Pakistan (June-July, 2018), identified and validated by Dr. Abdul Nazir, Assistant Professor, Department of Biotechnology, COMSATS University Islamabad, Abbottabad Campus, Pakistan. CHUA-112 is voucher code for the specimen in the herbarium, Department of Pharmacy, COMSATS University Islamabad, Abbottabad Campus, Pakistan. Fresh corms were chopped and dried under the shade at room temperature. Then the dry material was powdered, soaked in a (70%) aqueous methanol solution, with occasional shaking for fifteen, seven and three days. The macerate was filtered through a muslin cloth and then through a qualitative filter paper (Whatman, Grade 1).^22^ This process was repeated thrice. Then, a rotary evaporator (-760 mmHg at 37°C) was used to concentrate the liquid extract. The crude extract was analyzed phytochemically for all important constituents such as flavonoids, alkaloids, saponins, phenols and tannins.^23^

### Animals

All the experiments were performed in conformity with the guidelines from the Commission on Life Sciences, Institute of Laboratory Animal Resources, National Research Council^24^ and approved by the Ethical Committee. Sprague-Dawley (SD) rats were kept in the Animal House with food and water available *ad libitum*.

### Pharmacological investigations

#### Drugs and standards

Drugs and standards were purchased from the following sources: acetylcholine chloride, phenylephrine hydrochloride, atropine sulfate, pentothal sodium from Abbott Laboratories, Pakistan; while isoprenaline hydrochloride, potassium chloride, Nω-nitro-L-arginine methyl ester hydrochloride (_L_-NAME) and verapamil hydrochloride were obtained from Sigma chemicals company, USA.

#### *In vivo* studies

##### High salt-induced hypertensive rat (HSHR) model and grouping

SD rats (200-250 g) (n=60) were divided randomly into eight groups (n=5-7 in each group). The sampling was done by convenience sampling. Group 1 (normal control group) was given a normal diet. Group 2 (hypertensive group) was given NaCl (8% in diet + 1% in drinking water) for 8 weeks. Groups 3-5 (*S. guttatum-*treated group) were given NaCl (8% in diet + 1% in drinking water) and different oral doses of *S. guttatum* crude extract (100 mg/kg/day, 150 mg/kg/day and 300 mg/kg/day) once daily for 8 weeks. Groups 6-8 (standard treated group) were given oral daily doses of verapamil (5 mg/kg/day, 10 mg/kg and 15 mg/kg/day) along with a NaCl diet containing 8% NaCl + 1% NaCl in drinking water for 8 weeks.^25-27^

##### Invasive blood pressure recording in HSHR

The tracheal intubation of anesthetized (pentothal, 40–100 mg/kg, i.p) SD rats were performed using polyethylene tubing (PE-20). To monitor blood pressure, the right carotid artery was canulated using polyethylene tubing (PE-50) and affixed to a PowerLab Data Acquisition System (ADInstrument, Australia), through a pressure transducer (MLT 0699). An overhead lamp was used to maintain the animal's body temperature. Mean arterial pressure was monitored for 30 minutes in each group.^25,28^

##### Body weight profile

Body weight was determined at the beginning of the experiment in all groups and subsequently monitored weekly. After 8 weeks of treatment, the change in body weight was calculated.

#### *In vitro* studies 

##### Vascular reactivity studies

To investigate the crude extract-induced endothelium preservation effect in HSHR, we isolated aortae from the normotensive, hypertensive and treated groups. Aortic rings were hanged in tissue baths (10 mL), containing carbogen (5% CO_2_ and 95% O_2_) aerated normal Kreb's solution consisting of NaCl, 118.2 mM; KCl, 4.7 mM; MgSO_4_, 1.2 mM; KH_2_PO_4_, 1.3 mM; NaHCO_3_, 25.0 mM; Glucose, 11.7 mM; CaCl_2_, 2.5 mM, and kept at 37°C. The force was monitored by the PowerLab Data Acquisition System (ADInstrument, Australia) and a bridge amplifier (N12128) using a force displacement transducer (MLT 0201). Aortic rings were stabilized at 2 g isometric tension for 60-90 minutes by changing the Kreb's solution every 15 minutes. To determine the endothelial integrity, different concentrations of acetylcholine were used on phenylephrine (1 *µ*M) pre-constricted aortic rings.^25,28^

##### Isolated SD rat aortic preparations

Aortic rings were hanged in tissue baths filled with 10 mL of carbogen (5% CO_2_ and 95% O_2_) aerated normal Kreb's solution maintained at 37°C, affixed to a PowerLab Data Acquisition System (ADInstrument, Australia) and a bridge amplifier (N12128) using a force displacement transducer (MLT 0201). The rings were equilibrated for 60-90 minutes at an isometric tension of 2 g, while the solution was changed after every 15 minutes. Different concentrations (0.1–10 mg/mL) of *S. guttatum* were added to PE preconstricted rings. To determine the underlying mechanism, aortic rings were pre-treated with 1 *µ*M atropine or 10 *µ*M _L_-NAME for 30 minutes. In some experiments, endothelium-denuded rings from normotensive rats were used.^25,28,29^

##### Isolated right atrial preparations

The right atria from normotensive SD rats were dissected. The atrial preparations were hanged in tissue baths containing 10 mL of aerated Kreb's solution, maintained at 32°C, linked to PowerLab (ML 846) Data Acquisition System (ADInstrument, Australia) and bridge amplifier (N12128) via force transducer (MLT 0201). The tissues were stabilized at the resting tension of 1 g for 30 minutes. The muscarinic receptor involvement was studied in atropine (1 *µ*M) pre-treated atrial preparations.^25,28^

##### Statistical analysis

Data were normally distributed, as determined by the Shapiro-Wilk's test of normality. Data were expressed as mean ± standard deviation (SD) and the median effective concentrations (EC_50_ values) with the 95% confidence interval (CI). The % of change in MAP or body weight profiles were calculated by one-way analysis of variance (ANOVA) (followed by *post-hoc* Tukey HSD test). The % of vasorelaxation in normotensive and hypertensive rats were calculated by two-way ANOVA (followed by *post-hoc* Bonferroni test) using SPSS software, v. 21 (USA). The accepted level of statistical significance was set at 5%.

## Results

### Phytochemical constituents

Preliminary phytochemical analysis performed on *S. guttatum* corms extract indicated presence of alkaloids, flavonoids, phenols, phytosterols, saponins and tannins.

#### Pharmacological investigation

#### *In vivo* studies

##### Invasive blood pressure monitoring

The mean arterial pressure (MAP) values measured in different experimental groups are shown in Figures 1 and 2. The MAP of the HSHR group showed a 67.7% elevation in blood pressure as compared to the normotensive control group. This elevation in MAP was reversed by Sg.Cr treatment in a dose-dependent manner (p < 0.01) with up to 300 mg/kg concentration, where it reached its maximal MAP lowering effect (p < 0.001). The MAP in verapamil-treated rats (5 mg/kg and 10 mg/kg) also decreased in a dose-dependent manner (p < 0.01 and p < 0.001 respectively) reaching the maximal effect at a 15 mg/kg concentration (p < 0.001).

**Figure 1:** Mean arterial pressure in the normotensive, hypertensive and Sauromatum guttatum crude extract (Sg.Cr)-treated high salt-induced hypertensive rats at doses of 100 mg/kg, 150 mg/kg and 300 mg/kg (n=5-7; mean ± SEM). Compared with hypertensive control values, **p < 0.01 and ***p < 0.001

**Figure 2:** Mean arterial pressure in the normotensive, hypertensive and verapamil-treated high salt-induced hypertensive rats at doses of 5 mg/kg, 10 mg/kg and 15 mg/kg) (n=5-7; mean ± SEM). Compared with hypertensive control values, **p < 0.01 and ***p < 0.001

##### Body weight profile

High salt intake for 8 weeks caused a significant (p < 0.001) decrease in body weight in the hypertensive control group (Table.1). The treatment of hypertensive rats with Sauromatum guttatum crude extract (Sg.Cr) prevented significant changes in body weight at all doses, while verapamil-treated animals at 5 mg/kg showed a significant decrease in body weight (p < 0.05). Animals in the verapamil-treated groups (both 10 mg/kg and 15 mg/kg) did not show any significant changes in body weight (Table.1).

**Table 1:** Effect on body weight in normal control, hypertensive control and rats treated with different doses of the crude extract of Sauromatum guttatum (Sg.Cr) and verapamil. Values are expressed as mean ± SD (n=5-7)

Groups	Weight (g)	Weight (g) after 8 weeks
Normal control	244.66 ± 6.36	267.61 ± 3.08
Hypertensive group	249.28 ± 3.25	182.10 ± 5.09***
Sg.Cr 100 mg/kg treated	241.66 ± 3.81	245.01 ± 4.66
Sg.Cr 150 mg/kg treated	245.93 ± 6.43	250.90 ± 3.53
Sg.Cr 300 mg/kg treated	239.43 ± 1.48	248.63 ± 4.52
Verapamil 5 mg/kg treated	240.50 ± 1.41	214.23 ± 3.53*
Verapamil 10 mg/kg treated	242.25 ± 5.65	247.68 ± 2.96
Verapamil 15 mg/kg treated	245.83 ± 6.36	254.48 ± 3.32

Sg.Cr: Crude extract of Sauromatum guttatum. Values are expressed as mean ± SD (n=5-7).

* p < 0.05,

** p < 0.01 and

*** p < 0.001

vs. pretreatment values (One-way ANOVA analysis followed by Tukey HSD post-hoc test).

#### *In vitro* studies

##### *In vitro* vascular reactivity studies

In aortas isolated from the normotensive group, acetylcholine caused complete relaxation with an EC_50_ value of 0.2 *µ*M (0.1–0.3) (Figure 3). Aortas from hypertensive control rats, on the other hand, displayed only 5.5% acetylcholine-dependent relaxation as shown in Figure 3. *S. guttatum* crude extract treatment at doses of 100 mg/kg and 150 mg/kg partially restored the acetylcholine-induced relaxation to 38.5%, and 45.5%, respectively. However, rings from SD rats treated with 300 mg/kg *S. guttatum* crude extract showed 100% acetylcholine-dependent relaxation with an EC_50_ value of 0.6 *µ*M (0.3–1.0) (Figure 3). Treatment with 5 mg/kg verapamil caused only a negligible relaxation, while treatment with 10 mg/kg induced relaxation up to 16%. Interestingly, the increase in verapamil concentration up to 15 mg/kg did not further increase the acetylcholine-induced relaxation (Figure 3).

**Figure 3:** Typical tracings and graphs show the effect of acetylcholine (ACh) against phenylephrine-induced contractions in isolated rat aortic rings in normotensive, hypertensive control (A) and Sauromatum guttatum crude extract (Sg.Cr) treated high salt-induced hypertensive rats at doses of 100 mg/kg, 150 mg/kg and 300 mg/kg (B) and verapamil treated high salt-induced hypertensive rats at doses of 5 mg/kg, 10 mg/kg and 15 mg/kg (C) (n=5-7; mean ± SD). Compared with hypertensive control values, *p < 0.05, **p < 0.01 and ***p < 0.001.

##### *In vitro* rat aorta studies

Pharmacological studies were performed in normotensive rat aortae to investigate the antihypertensive effect of the *S. guttatum* crude extract. Relaxation induced by the cumulative addition of the crude extract on PE-preconstricted aortic rings showed an EC_50_ value of 0.15 mg/mL (0.10-0.20) (Figure 4). _L_-NAME (10 *µ*M) pre-treated rings showed relaxation with EC_50_ value of 5.1 mg/mL (3.0-7.1) (Figure 4). *S. guttatum* crude extract failed to induce relaxation in atropine (1 *µ*M) pretreated and endothelial denuded rings. Both atropine pretreatment (1 *µ*M) and endothelium removal decreased the crude extract-induced relaxation by 26% and 14%, respectively (Figure 4). *S. guttatum* crude extract also produced vasorelaxation in aortic rings preconstricted with high K^+^ concentration, with an EC_50_ value of 9.03 mg/mL (8.06-10.00). In comparison, verapamil relaxed the high K^+^ preconstricted aortas with EC_50_ value of 2.02 *µ*M (1.02-3.02) (Figure 5).

**Figure 4:** Tracing (A) and graph (B) show the effect of Sauromatum guttatum crude extract on intact, L-NAME (10 *µ*M) and atropine (1*µ*M) pretreated and endothelium denuded normotensive rat aorta against phenylephrine-induced contractions (n=5-7; mean ± SD). *p < 0.05, **p < 0.01 and ***p < 0.001 vs Control (pretreated values). Two-way ANOVA analysis followed by Bonferroni's post-hoc test.

**Figure 5:** Tracing (A) and graph (B, C) show the effect of Sauromatum guttatum crude extract on high potassium (K+) (80 mM)-induced contractions in intact rat aortic preparation (n=5-7; mean ± SD). *p < 0.05, **p < 0.01 and ***p < 0.001 vs. Control (pretreated values). Two-way ANOVA analysis followed by Bonferroni's post-hoc test

##### *In vitro* rat right atrial study

Right atrial strips from normotensive rats were used to investigate chronotropic and inotropic effects of *S. guttatum*. The crude extract showed a dose-dependent decrease in force of contraction and heart rate with an EC_50_ value of 2.99 mg/mL (1.08-4.90) and 1.83 (1.02-2.64), respectively (Figure 6). In atropine pre-treated tissues, the decrease in force of contraction and heart rate was shown to be 29% and 44%, respectively (Figure 6).

**Figure 6:** Tracing (A) and graph (B, C) show the inotropic and chronotropic effects of Sauromatum guttatum crude extract without and with atropine (1 µM) pre-treated normotensive rat right atria (n=5-7; mean ± SD). *p < 0.05, **p < 0.01 and ***p < 0.001 vs. control (pretreated values). Two-way ANOVA analysis followed by Bonferroni's post-hoc test.

## Discussion

Traditionally, *S. guttatum* has been used in the management of blood disorders. It contains ample amounts of magnesium and potassium.^11,15^ Additionally, it has been reported as an antioxidant, spasmolytic and Ca^2+^ entry blocker agent.^19^^,^^20^ Plants with antioxidant properties, DASH diet rich in potassium and magnesium and Ca^2+^ entry blockers are recommended for hypertension management.^8,30,31^ The present study, using the hypertensive SD rat model, was carried out to explore the use of the crude extract from *S. guttatum* as a potential antihypertensive drug. Different doses of *S. guttatum* crude extracts were given orally to high salt diet-induced hypertensive SD rats. This treatment resulted in a significant decrease in the mean arterial pressure, with a maximum effect observed at the dose of 300 mg/kg. This effect of the crude extract was comparable to that of verapamil, which is a standard antihypertensive drug and calcium channel blocker.^31^ This finding revealed that *S. guttatum* extract is effective against the development of high salt diet-induced experimental hypertension. However, further studies were needed to identify the possible underlying action mechanism.

Since blood pressure is the product of elevated peripheral vascular resistance and high cardiac output,^32^ further experiments were carried out using isolated vascular and cardiac preparations. First, an attempt was made to establish how high salt intake induces endothelial dysfunction. Endothelial integrity was confirmed by applying sub maximum concentrations of acetylcholine on phenylephrine-preconstricted aortic rings from HSHR. Acetylcholine failed to induce relaxation in aortic rings in the HSHR group, indicating that the endothelium was damaged. This finding is supported by previous studies.^33-35^ In aortic rings from normotensive rats, on the other hand, same concentrations of acetylcholine induced relaxation, indicating the presence of a functional endothelium. In the extract-treated groups, the response to acetylcholine was restored. These results indicate that the crude extract treatment can reverse the endothelial damage and also prevent the elevation in mean arterial pressure observed in *in-vivo*. In comparison, verapamil failed to induce vasorelaxation in aortic rings in HSHR control or treated rats, indicating that its action mechanism is different from that of the crude extract. *S. guttatum* extract exerts its antihypertensive function on experimental hypertension by partially preserving the endothelial function.

Further *in vitro* studies were carried out in the aorta to investigate the underlying action mechanism(s). In acetylcholine-preconstricted aortic rings of normotensive rats, the cumulative additions of crude extract concentrations induced vasorelaxation. Endothelial denudation completely reversed this effect, suggesting that vascular endothelial-derived factors might play a role. However, high concentrations of acetylcholine still induced relaxation, suggesting the involvement of different mechanisms. To study the involvement of nitric oxide, aortic rings were pretreated with _L-_NAME, a nitric oxide synthase inhibitor.^36^ Interestingly, the vasorelaxant effect of *S. guttatum* extract was reduced by about 75% at 1 mg/mL concentration, while higher concentrations shifted the response curve to the right. These findings suggest that *S. guttatum* induces vasorelaxation through both an endothelium-dependent (at a lower concentration) and endothelium-independent (at a higher concentration) pathways. The endothelium-dependent component could be attributed to nitric oxide. In vascular endothelial cells, nitric oxide release is coupled to muscarinic receptors.^37^ To see if the effect of *S. guttatum* crude extract is linked to muscarinic receptors and nitric oxide, aortic rings were precontracted with atropine, a muscarinic receptor antagonist.^37^ This pretreatment abolished vasorelaxation associated to the crude extract of *S. guttatum*, thus indicating an action through a muscarinic receptor-linked NO pathway. Atropine or _L-_NAME failed to inhibit relaxation at higher concentrations of the crude extract, further suggesting that the extract might also act on vascular smooth muscles. To test this hypothesis, aortic rings were precontracted with high K^+^ concentration. Interestingly, the cumulative addition of the crude extract induced a vasorelaxant effect that was 10 times less potent than against PE. High K^+^ was used to induce contractions, as it activates voltage-dependent calcium channels (VDCs) and Ca^2+^ release through depolarization, resulting in vasoconstriction.^38,39^ These findings indicate that the crude extract of *S. guttatum* also inhibit Ca^2+^ entry through VDCs. It also suggests that vascular NO plays a dominant role in the vasorelaxant and antihypertensive effects of *S. guttatum,* in addition to the effect on vascular smooth muscles.

To investigate the effect of *S. guttatum* extract on cardiac parameters, isolated rat atrial strips were used. *S. guttatum* showed negative inotropic (82%) and chronotropic (56%) effects when added cumulatively to spontaneously contracting right atrial strips. To test the possible role of cardiac muscarinic receptors, atrial strips were pretreated with atropine. This pretreatment partially inhibited the effect of the crude extract of *S. guttatum*, thus indicating a possibility that the observed negative inotropic or chronotropic effect is due to cardiac muscarinic receptor activation. However, our findings revealed that the extract is more selective for the vascular than cardiac muscarinic receptors.

*S. guttatum* was also tested for the presence of phytochemical constituents. It was found to contain flavonoids, phenols, and tannins. Previous studies revealed the therapeutic effect of flavonoids, phenols and tannins on hypertension.^40-42^ So, these constituents might be the active agents responsible for lowering blood pressure and the vascular effects in high salt-induced hypertension. Future phytochemical studies will be focused on isolating the active components and exploring the underlying mechanisms, such as calcium blocking and nitric oxide pathway at the molecular level.

## Conclusion

These findings indicate that *S. guttatum* has antihypertensive activity resulting from the vasodilatory and atrial myocardium depressant effects linked to muscarinic receptors. Endothelial function preservation, muscarinic receptor dependent NO release and Ca^+2^ movement inhibition are the underlying mechanisms of vasodilation. *S. guttatum* also exerts negative inotropic and chronotropic effects, possibly due to activation of cardiac muscarinic receptors. Our results observed in the SD rat model provide pharmacological explanation for *S. guttatum* antihypertensive potential.
